# Use of Nelaton’s Catheter

**Published:** 1876-05

**Authors:** T. C. Wallace

**Affiliations:** Cambridge, N.Y.


On the Use of Nelaton's Catheter in Stricture of the Urethra, Enlarged Prostate, etc.—*By T. C. Wallace, M.D., of Cambridge, N. Y.*—For the benefit of my professional brethren who are often sorely puzzled, and who sometimes find it impossible to introduce the catheter, by reason of stricture of the urethra, enlargement of the prostate, or perhaps from a swollen and bruised state of the parts, caused by too vigorous use of instruments, I desire to report the invariable success which has thus far attended the use of the India-rubber catheter in my hands.

Nearly two years ago I was in attendance upon an elderly, gentleman who required daily catherization. Each introduction of the instrument was provoking increased suffering and becoming more difficult of accomplishment, and I was convinced the time was not far distant when it would be found impossible to enter the bladder with any ordinary catheter.

I procured one of Squire's prostatic catheters, which at first seemed an improvement on the silver and gum-elastic ones previously used; but after a few times using I could not introduce it as readily as I could a silver one. My attention was then called to the India-rubber instrument, and I procured a Nelaton's soft gum catheter. When I got it, it seemed to me utterly improbable (and well nigh impossible) that an instrument possessing so little firmness, and so entirely limp, would ever pass through the urethra and enter the bladder. However, I was most agreeable disappointed, for its passage was attended with no difficulty whatever, and with scarcely any pain to my patient—not a tithe of what accompanied the use of the other. Since that time I have used this form of catheter altogether, entirely discarding all others. It has never disappointed me, neither have I ever found any difficulty in its introduction. Last week I was called to see a man living some distance from here, who was suffering intensely from retention of urine, caused by prostatic hypertrophy. I found that three of the ablest physicians had been trying for twenty-four hours to catheterize him without success. The case was desperate, and puncture of the bladder through the rectum had been determined upon, and would have been resorted to, but that the patient would not consent to it until my arrival.

The Nelaton catheter was introduced into the bladder in less than one minute, producing very little pain. I left one with the patient, and he has since been able to relieve himself whenever necessary, introducing it as readily as could the most skilful surgeon. My only object in writing the above is to disseminate through the columns of the *Journal* a wider knowledge of this invaluable instrument, which has only to be used to be thoroughly appreciated, and which is a priceless boon as well to the patient as to the physician.—*Amer. Journal Med. Sciences.*

